# Enhanced Mechanical Stability and Hydrophobicity of Cellulose Aerogels via Quantitative Doping of Nano-Lignin

**DOI:** 10.3390/polym15051316

**Published:** 2023-03-06

**Authors:** Xiaoyu Wang, Xinyu Yang, Zhen Wu, Xiaoyan Liu, Qian Li, Wenkai Zhu, Yetao Jiang, Lei Hu

**Affiliations:** 1 Jiangsu Key Laboratory for Biomass-Based Energy and Enzyme Technology, School of Chemistry and Chemical Engineering, Huaiyin Normal University, Huaian 223300, China; 2 College of Chemistry and Materials Engineering, Zhejiang A&F University, Hangzhou 311300, China

**Keywords:** cellulose nanofibers, lignin nanoparticles, mechanical enhancement, glue agent, hydrophobic modification

## Abstract

As a porous biomass sustainable material, cellulose aerogel has attracted significant attention due to its unique properties in various applications. However, its mechanical stability and hydrophobicity are huge obstacles hindering practical applications. In this work, nano-lignin quantitative doping cellulose nanofiber aerogel was successfully fabricated via liquid nitrogen freeze drying combing vacuum oven drying. The impact of various parameters (lignin content, temperature, and matrix concentration) on the property of the as-prepared materials was systematically explored, revealing the optimum conditions. The morphology, mechanical properties, internal structure, and thermal degradation of the as-prepared aerogels were characterized by various methods (compression test, contact angle, SEM, BET, DSC, and TGA). Compared with pure cellulose aerogel, the addition of nano-lignin did not significantly change the pore size and specific surface area of the material but could improve its thermal stability. In particular, the enhanced mechanical stable and hydrophobic properties of cellulose aerogel via the quantitative doping of nano-lignin was confirmed. The mechanical compressive strength of 160–13.5 C/L-aerogel is as high as 0.913 MPa, while the contact angle was nearly reaching 90°. Significantly, this study provides a new strategy for constructing a novel cellulose nanofiber aerogel with mechanical stability and hydrophobicity.

## 1. Introduction

Aerogel is a highly dispersed solid material composed of colloidal particles or polymers with a nano-porous network structure. [1]. Due to their unique structural characteristics, aerogels have a wide range of potential applications [2,3]. They are expected to have excellent performances as supercapacitors [4], gas sensors [5], energy storage [6], thermal and acoustic insulation [7], catalyst supports [8], and absorbents [9], while the main components of traditional aerogel materials are silicon materials and metal oxides [10,11,12]. Aerogels can be classified according to their composition. Generally, three types of aerogels are described: inorganic aerogels, organic aerogels, and inorganic–organic hybrid aerogels [13]. For the past few years, many attempts have been made to fabricate aerogels from new ingredients such as graphene, carbon nanotubes, and cellulose [14,15,16,17,18].

Cellulose is currently the most common linear polymer consisting of glucose monomers in the world [19]. Cellulose nanofibers obtained from renewable resources have attracted increasing interest due to their high aspect ratio, low mass density (~1.5 g/cm^−3^), biodegradability, and minimal ecological impact [20,21]. Cellulose aerogels, especially for the nanocellulose aerogels consisting of native cellulose I crystals with a very high modulus and strength, are easily compressed to a larger strain of more than 50% without disintegration. With respect to inorganic aerogels, it is generally less brittle and even flexible [22]. As the third generation of new materials, cellulose aerogels can be used not only as a pharmaceutical carrier but also as a template material. Cellulose aerogel is a promising adsorption material for water purification because of its network structure, while it can also be used as nano-porous scaffolds for tissue engineering. Cellulose aerogels can be fabricated from either homogeneous cellulose solution [23,24] or heterogeneous aqueous nanocellulose suspensions [25,26]. Aerogels prepared from cellulose solutions require a long time and multiple steps to dissolve cellulose in the solvent, rather than the amorphous or cellulose II structure at dissolution, and supercritical or freeze-dried nanocellulose aqueous suspensions have the advantage of retaining the Iβ crystal structure of native cellulose [27]. Cellulose aerogels have been proved, but so far, the emphasis has been only on chemical modification, regenerated cellulose, and in micro fibrillated cellulose–starch blends. However, if cellulose is derivatized or regenerated, the mechanical integrity of the aerogels is reduced because they lose the favorable cellulose I crystal structure [27].

The successful production of CNF-based aerogels has been demonstrated in several studies. In addition, freeze-drying and supercritical carbon dioxide drying are the most effective methods for removing water or other solvents without collapse due to capillary effects. However, because the cohesion of CNF aerogels is based on hydrogen bonding and physical entanglement, its structural stability will inevitably be lost when it meets water, which is due to its diffusion in the CNF network and the destruction of cellulose–cellulose hydrogen bonds. The chemical crosslinking of cellulose aerogels has proved to be a successful choice to improve their structural stability in aqueous media by involving hydroxyl groups [28,29]. The outstanding characteristics of hydrazone crosslinking chemistry are a fast gelation time and low toxicity, which provides the material with good mechanical properties without any type of external stimulus or initiator to form bonds [30]. In fact, some materials with a strong interaction with the cellulose matrix may also have a reinforcement effect. PEG containing ether oxygen atoms with a different molecular weight were used to form a hydrogen bond interaction with the cellulose chain, which could increase the tensile strength of cellulose gel [31]. In addition, the evenly dispersed CNFs and PVA further strengthened the CNF/PVA hybrid aerogel by crosslinking the hydroxyl groups on CNF and PVA with glutaraldehyde [1]. Recently, cellulose acetate and cellulose acetate butyrate have been used as precursors for organic networks to prepare aerogels by supercritical CO_2_ drying. Note that cellulose derivatives require the addition of crosslinking agents to stabilize aerogel networks due to the reduction in or total elimination of hydroxyl groups [29].

Lignin, next only to cellulose, is the second most abundant biopolymer and the main source of aromatic structures in the world [32]. They are generally distributed with hemicelluloses in the spaces of inter cellulose microfibrils in primary and secondary walls, as well as in the intermediate lamellae, as cementing components connecting the cell to the sclerotic cell wall [32]. Lignin is more nonpolar than cellulose and acts as a chemical adhesive within and between cellulose fibers [32]. Instead of treating lignin as a waste, it can be used to enhanced composites, carbon fibers, plastics, and nanomaterials to expand the economic and environmental benefits of materials [33,34]. Different lignin nanoparticles can be obtained by chemical, physical, and mechanical treatment selecting different lignins as raw materials, including Kraft, acetylated, phosphorylated, etc. In some recent studies, lignin particles were used as a nanofiller to strengthen polysaccharides, proteins, natural rubber, and synthetic polymers [35]. Additionally, the presence of lignin as a reinforcement or as a matrix in different polymer nanocomposites can promote the improvement of the mechanical strength, thermal stability, antibacterial activity, the adsorption of Pb(II) ions, oil-spill clean-up processes packaging, and other properties [36]. Since the surface functional groups of cellulose nanofibers are rich and easy to modify, it is a solid amine grafting carrier that has attracted much attention recently. Most solid amine adsorbents are supported by inorganic porous materials, which have a high cost, difficulty in regeneration, and may produce secondary pollution, limiting their application in industry. Therefore, the development of green pollution-free and renewable biobased carriers has become a research hotspot [37,38]. The construction of cellulose aerogels with a three-dimensional network structure and high specific surface area without affecting the amino loading on the surface of cellulose can greatly improve the ability of dealing with gases. However, due to the random crosslinking of the precursor scaffold and the natural hydropathy of the surface hydroxyl group, the stability and amino grafting rate of these aerogels are poor, which limits their functional development and application.

In this work, simple routes for nano-fibrillated cellulose aerogel with water-resistant and promoted mechanical properties are introduced. No crosslinking agent is required in the process; not only do the preserved intramolecular hydrogen bonding and the entangled, long, partly amorphous nanofibers act as crosslinkers themselves, but the existent lignin particles work as glue agents. Furthermore, lignin, as a complex three-dimensional polymer, is thermally more stable than cellulose and hydrophobic in nature. Therefore, it can promise cellulose-based porous materials mechanical strength in wet conditions and displays a superior thermal stability. Furthermore, this study not only provides a new perspective on the utilization of lignin components, but also does not go against the natural green property of cellulose aerogel as an adsorption material, which is expected to provide a theoretical basis and technical support for the development of cellulose aerogel as a green and efficient carbon dioxide adsorption material.

## 2. Materials and Methods

### 2.1. Materials

Cellulose nanofibrils (CNF, solids 2.95%) were purchased from the University of Maine Process Development Center (Orono, ME, USA). It was prepared mechanically using a pilot-scale double disk refiner to defibrillate a bleached softwood kraft pulp. The chemical composition of cellulose is 83.3% cellulose, 14.7% hemicellulose, and less than 0.5% lignin. The diameter of the CNF was in the range of 20–50 nm, while its length was from 600 to 1000 nm, and it had a crystalline of 75%. Lignin (Dealkaline, L0045, TCI, Portland, OR, USA) was purchased from Tokyo Chemical Industry. Cross-linker KymeneTM resin (Ashland Hercules Inc., Hopewell, VA, USA) was purchased and added to the nanofibril cellulose suspension. Distilled water was used for all preparations.

### 2.2. Preparation

#### 2.2.1. Preparation of Nano-Lignin

The purchased lignin was soaked in distilled water at a solid content of 2 wt% for 24 h. It was nano fibrillated by passing through a supermass colloider (Model: MKCA6-5JR, Masuko Sangyo Co., Ltd., Kawaguchi-City, Japan) at a rotor speed of 1500 rpm. Grinding treatment was performed with a clearance gauge of −2.0, corresponding to a 0.25 m shift from the 0 position, which was determined as the point of slight contact between the two grinding stones. The presence of lignin between the two stone disks ensured that there was no direct contact between the disks. Finally, the nano-lignin was prepared.

#### 2.2.2. Preparation of CNF/Nano-Lignin Aerogels

A mixture of 1.5% CNF aqueous suspensions and lignin (10 wt% of dry CNF) was poured into an open tube (1.5 inches in length and 0.81 inches in diameter) and sealed with aluminum foil. The samples were flash frozen in liquid nitrogen for 1 min and lyophilized in a vacuum lyophilizer (Labconco, Inc., Kansas City, MO, USA) at −51 °C for 2 days. A columnar aerogel was obtained. The samples were oven heated at 160 °C for 2 h to promote lignin melting to form 3D networks. Finally, CNF-lignin aerogel was prepared.

### 2.3. Characterizations

#### 2.3.1. Measurement of Density

The bulk density of the nanocellulose aerogel was determined by the following formula: ρ = m/V, where m and V denote the weight and volume, respectively.

#### 2.3.2. Compression Properties

The compressive modulus and compressive strength were measured at a strain rate of 0.2 mm min^−1^ on an INSTRON 5567 universal testing machine (Canton, MA, USA) using a 50 kg load sensor.

#### 2.3.3. Scanning Electron Microscopy (SEM)

A Zeiss Auriga SEM/FIB (Zeiss, Germany) rossbeam workstation was used to analyze the surface morphology of the nano-cellulose aerogels. The sample was coated with gold–palladium and operated at 20 kV.

#### 2.3.4. DSC Analysis

The thermal properties of the products were measured using a TA Q2000 (USA) differential scanning calorimeter. The DSC specimens (about 5 mg) were sliced from the prepared aerogels. To prevent oxidation, 2 temperature cyclic scans were carried out at a nitrogen flow rate of 100 mL/min. The first scan removes all residual moisture at a heating rate of 10 °C/min and erases any thermal history from 30 to 300 °C; the specimen was stored at 300 °C for 5 min. It was then cooled to 30 °C at a rate of 10 °C/min and reheated to 300 °C at a rate of 10 °C/min.

#### 2.3.5. Thermogravimetric (TG) Analysis

Thermogravimetric analysis was performed on Pyris 1 TGA (PerkinElmer, Waltham, MA, USA). The scanning temperature of nano cellulose aerogel (about 5 mg) was 35~600 °C and the heating rate was 20 °C/min as the flow rate of nitrogen was 20 mL/min to avoid sample oxidation.

#### 2.3.6. N_2_ Sorption Isotherms

N_2_ adsorption isotherms were obtained at 77 K by a Micrometrics ASAP 2020 analyzer (Micrometrics, Norcross, GA, USA). The calculation of the specific surface area and average pore size were, respectively, obtained by the BET and BJH equation. Nano-cellulose aerogels were placed in a vacuum overnight at room temperature before the analysis.

## 3. Results

### 3.1. Hydrophobic Stability

**Figure 1:**
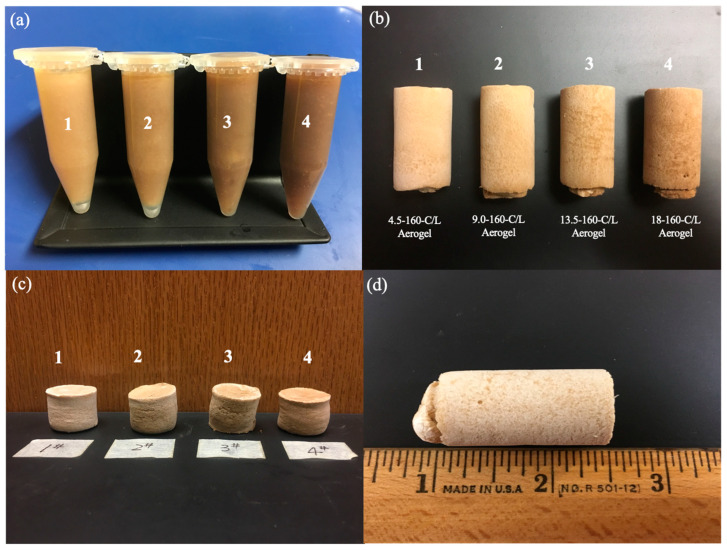
Photographic images of (**a**) CNF/nano-lignin mixture, (**b**) CNF/nano-lignin aerogel (at different nano-lignin values 4.5 wt%, 9.0 wt%, 13.5 wt%, and 18 wt%) and (**c**) CNF/nano-lignin aerogels after compression test and (**d**) CNF/nano-lignin aerogel (at nano-lignin value of 4.5 wt%).

Representative photographs are shown in the CNF/nano-lignin mixture (Figure 1a), CNF/nano-lignin aerogel (Figure 1b), CNF/nano-lignin aerogels after compression test (Figure 1c), and CNF/nano-lignin aerogel at a nano-lignin value of 4.5 wt% (Figure 1d). It has been shown that the macroscopical effect of the addition of lignin to the CNF/nano-lignin aerogel is only shown in color. The color of the CNF/nano-lignin aerogels changed from light brown to dark brown with the increase in the lignin concentration. After lignin was mechanically added into the nano particles, it was evenly dispersed in aqueous solution. Figure 1c is the picture of the CNF/nano-lignin aerogel after a mechanical compression test. Although the aerogels have little rebound after compression, it still maintained the complete structure and there exists no collapse or fracture.

**Figure 2:**
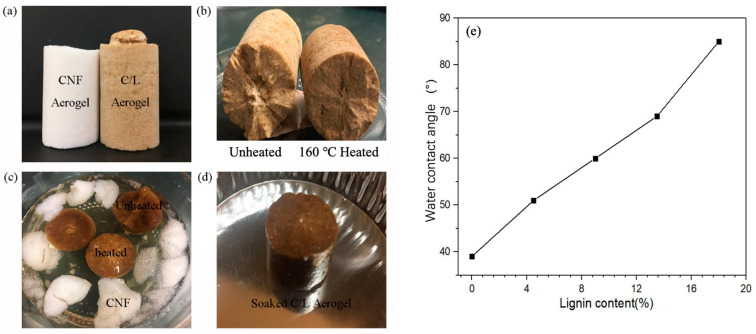
Photographic images of (**a**) CNF Aerogel and CNF/nano-lignin aerogel; (**b**) CNF/nano-lignin aerogels before and after heat treatment; (**c**) CNF and CNF/nano-lignin aerogels soaked in water for two hours; (**d**) CNF/nano-lignin aerogel after soaking; (**e**) contact angle of CNF aerogel and CNF/nano-lignin aerogels.

As displayed in Figure 2a, the added nano-lignin did not interfere with the formation and construction, but it did interfere with the color of the aerogel. Figure 2b shows the cross-section of the CNF/nano-lignin aerogels before and after heating in a vacuum oven. Moreover, the cross section of the heated aerogel was more smooth and complete, and an exquisite appearance was displayed. The CNF and CNF/nano-lignin aerogels were immersed in distilled water for 2 h as shown in Figure 2c. Upon contact with water, the CNF aerogel quickly absorbed water and swelled, and its columnar structure collapsed and eventually dispersed in distilled water. However, the CNF/nano-lignin aerogels maintained their original shape regardless of heating. In particular, the heated CNF/nano-lignin aerogel was taken out as a whole after soaking for a certain time, and its original macroscopic morphology was maintained, as shown in Figure 2d. The addition of the nano-lignin results greatly improved the water resistance of the CNF/nano-lignin aerogels; the contact angle increased linearly with the increase in the lignin content, as shown in Figure 2e. Moreover, the vacuum heat treatment better enhanced the structural stability of the material. Furthermore, the amount of nano-lignin added had an impact not only on the waterproof performance but also on the mechanical strength.

### 3.2. Compression Properties and Density

**Figure 3:**
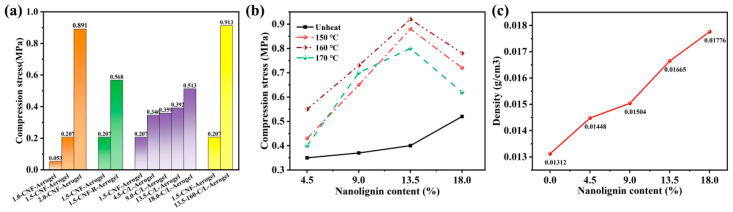
Compression properties of prepared aerogels. (**a**) Compression stress of 1 wt% CNF-aerogel, 1.5 wt% CNF-aerogel, 1.5 wt% CNF-aerogel with resin, 2 wt% CNF-aerogel, and 1.5 wt% 160-CNF/lignin-aerogel. (**b**) Compression stress of CNF/lignin-aerogel treated with different temperatures (not heated, 150, 160, and 170 °C). (**c**) Densities of prepared aerogels of different lignin content (0, 4.5 wt%, 9 wt%, 13.5 wt%, 18 wt%).

Figure 3a shows the mechanical strength of the CNF aerogels increased with the increasing cellulose concentration, which follows that the mechanical compressive strength of aerogel can be improved by increasing the concentration of the aerogel matrix material. This can be interpreted as the cross-linking points increased as an improvement in the cellulose nanofibers mass fraction [39]. When adding 5% of fiber dry weight polyamide- epichlorohydrin resin into 1.5-CNF-aerogel, because the crosslinking agent contains a quaternary ammonium group, it can be attached to negatively charged fibers and can be used to enhance the bonding strength between CNF fibers. The mechanical strength of aerogel increased with the addition of lignin, and its strength improved with the increase in the amount of lignin. Furthermore, the heated CNF/nano-lignin aerogel showed an excellent mechanical strength compared with CNF/nano-lignin aerogels with no heat treatment; specifically, the aerogels heated under 160 °C shown in Figure 3b displayed the best mechanical property. In Figure 3c, the density of the CNF/nano-lignin aerogels increased as the amount of lignin increased.

In the process of cell formation, lignin is a polymer deposited in the cell wall, and it interpenetrate the fibers to strengthen the cell wall [40]. Rather than treating lignin as waste, lignin can be used to strengthen the composite materials, as well as play the role of an adhesive and reinforcement. Particularly, the mechanical compressive strength of 160–13.5 C/L-aerogel as high as 0.913 MPa in our research is better than those of previously reported aerogels [39]. It can be seen that adding the appropriate amount of lignin nanoparticles can improve the mechanical properties of nanocellulose aerogel and enhance the internal structural framework of the aerogel.

### 3.3. DSC Analysis

**Figure 4:**
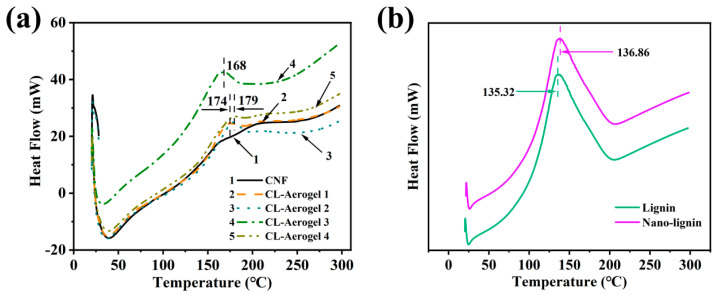
DSC curves of prepared aerogels of different lignin content (**a**),initial and nano-lignin (**b**) (0, 4.5 wt%, 9 wt%, 13.5 wt%, 18 wt%).

According to Figure 4, the initial softening temperature of the lignin nanoparticles was 137 °C, while that of cellulose was over 170 °C. When lignin nanoparticles are mixed with CNF, the softening temperature of the CNF aerogel was around 170 °C. The introduction of lignin has no significant effect on the glass transition temperature of cellulose aerogel. Therefore, the introduction of lignin does not affect the thermal stability of cellulose aerogel materials. When lignin nanoparticles are mixed with CNF, the softening temperature increases to about 170 °C. As shown in Figure 3b, the variation in the mechanical compressive strength of the CNF/nano-lignin aerogels at 150, 160, and 170 °C was obtained by a vacuum high-temperature treatment in the temperature range of 140–180 °C. The mechanical compressive properties of the CNF/nano-lignin aerogels heated in a vacuum oven are obviously better than that of CNF/nano-lignin aerogels without heating. On one hand, the mechanical compressive properties of the CNF/nano-lignin aerogels increased with the increasing lignin nanoparticle content in the aerogels without heating. On the other hand, the mechanical compressive properties of the CNF/nano-lignin aerogels heated in a vacuum oven increased first and then decreased with the increase in the lignin content. When the lignin nanoparticle content was 13.5 wt% of cellulose dry weight, the mechanical compressive strength of the CNF/nano-lignin aerogel reached the maximum value of 0.913 MPa. The lignin particles in the CNF/nano-Lignin aerogel are similar to carbon black particles [18]. The abundant hydroxyl group in lignin can form a hydrogen bond with the hydroxyl group in cellulose, thus showing a good reinforcing ability. As a thermoplastic polymer, lignin can flow and deform when heated and maintain a certain shape after cooling [19]. In a certain temperature range, lignin was softened by heating and hardened by cooling to play the role of an adhesive.

### 3.4. SEM Analysis

**Figure 5:**
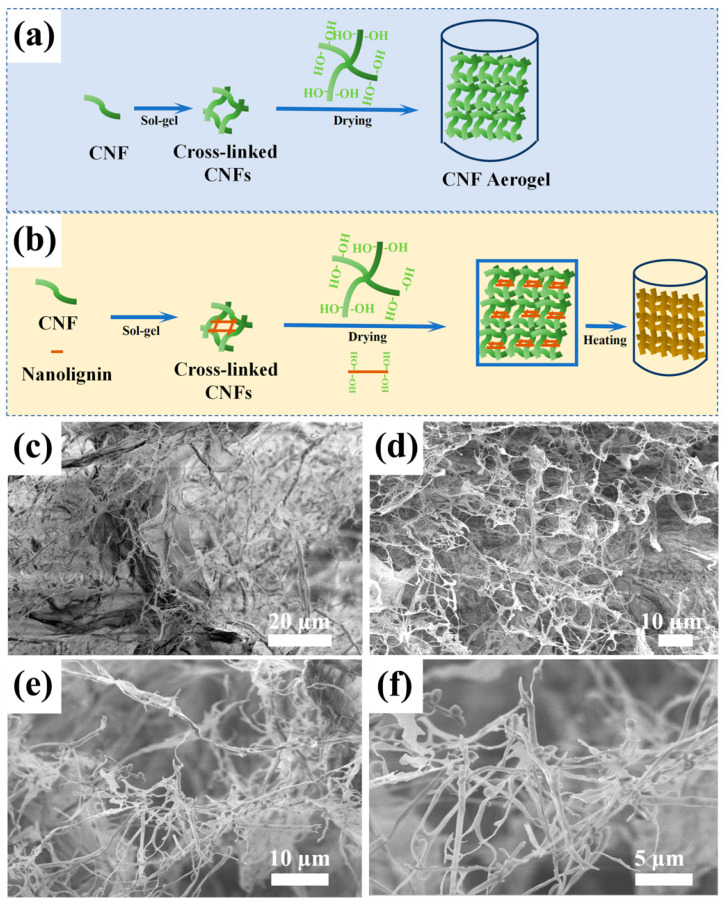
(**a**,**b**) Schematic of the crosslinking of the CNF and CNF/nano-lignin aerogels. CNF SEM images of prepared 160-CNF/nano-lignin aerogels. (**c**,**d**): ×2000; (**e**): ×5000; (**f**): ×10,000.

The internal structure of the prepared 160-CNF/nano-lignin aerogels was investigated by scanning electron microscopy. Figure 5a,b shows the fabrication mechanism and formation procedure of the CNF and CNF/nano-lignin aerogels. The CNFs were crosslinked to form the corresponding gel using the sol-gel method. Thereafter, the CNFs were placed for further cross-linking and the CNF aerogels were prepared via a liquid nitrogen freeze drying method. The molecules of nanocellulose in CNF aerogels interacted and intertwined because of the hydrogen bonding force mainly.

Figure 5c displayed spherical dark particles on the fibers of the 160-CNF/nano-lignin aerogels, and some of the fibers are glued together by dark materials. According to the results in Figure 4b, the softening temperature of nano-lignin particles was about 137 °C. As a thermoplastic polymer material, lignin can be softened by heating and hardened by cooling. When the CNF/nano-lignin aerogel was treated using a vacuum oven at 160 °C, dark lignin nanoparticles were clearly visible on the surface of cellulose in the microstructure of the 160-CNF/nano-lignin aerogels, and the fiber branches were coated with lignin nanoparticles as well. The “branch” structure is relatively strong, thus providing a high-strength skeleton structure for lignin nanoparticles, while the lignin acted as an adhesive filling the pores between the cellulose fibers. Furthermore, the internal structure of the 160-CNF/nano-lignin aerogels showed a regular honeycomb network structure, which is not found in the microstructure of pure CNF aerogel. It can be seen that the addition of lignin makes the internal structure of cellulose aerogel tend to be regular.

### 3.5. Thermo Gravimetric (TG) Analysis

**Figure 6:**
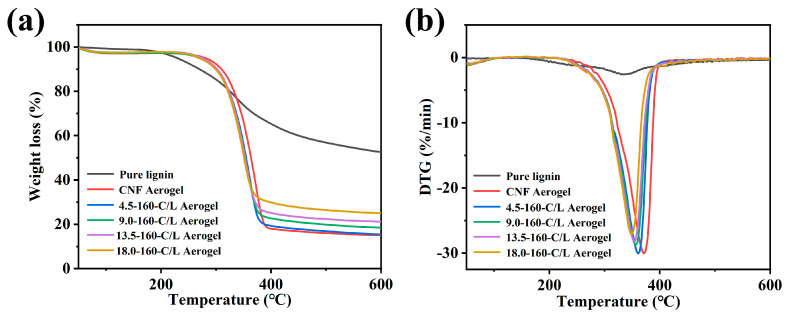
TG (**a**) and DTG (**b**) curves of pure lignin, CNF aerogel and CNF/nano-lignin aerogels.

As a complex three-dimensional polymer, lignin is more stable than cellulose and hemicellulose. Therefore, the difference in the lignin content is bound to have a certain influence on the thermal stability of the CNF/nano-Lignin aerogel. The curves for the thermogravimetric analysis of all cellulose aerogel samples are shown in Figure 6. TG curves of all the samples were divided into three weight-loss stages, including the free water removal stage, volatile analysis stage, and deep pyrolysis stage. The main pyrolysis temperature of lignin was in the range of 150–650 °C, and the main gas phase products included CO, CO_2_, and CH_4_. Because the basic structure of lignin is phenylpropane, the benzene ring has a methoxy group, and the carbon frame that constitutes the structural unit of phenylpropane is generally used as the basic in units, and the C content of lignin is up to 60–66%. With the increasing lignin nanoparticles in the CNF/nano-lignin aerogel, as the carbon residue of the aerogel sample became higher, the initial pyrolysis temperature moved to the high side while the maximum weight loss peak moved to the left. The three-dimensional network macromolecule of lignin made its thermostability better than that of hemicelluloses and cellulose [41].

### 3.6. N_2_ Sorption Isotherms

**Figure 7:**
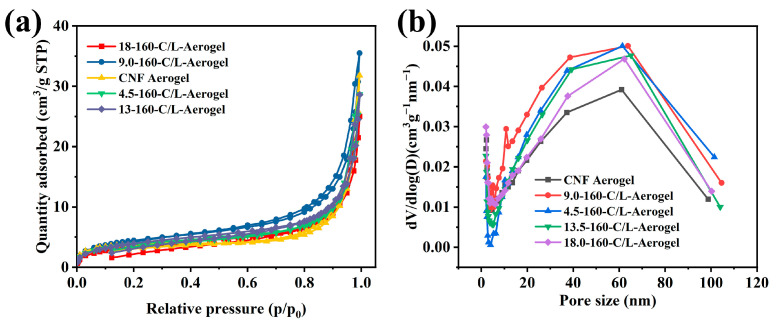
Analysis of N_2_ sorption isotherms and pore size distribution. (**a**) N2 sorption isotherms at 77 K of the CNF/nano-lignin aerogels; (**b**) pore size distributions of the CNF/nano-lignin aerogels.

Figure 7 exhibits N_2_ sorption isotherms and the pore size distribution of CNF/nano-lignin aerogels measured at 100 °C. It can be seen that aerogels with different contents of nano-lignin particles have similar rules of adsorption–desorption as shown in Figure 7a. At the low-pressure part of the adsorption curve (0–0.1), the nitrogen adsorption capacity increased slowly at a variable speed, and the intermediate pressure section (0.2–0.8) increased linearly, while during the high-pressure section (0.8–1.0), it increased rapidly. This is consistent with the characteristics of Langmuir II adsorption isotherms. In the high-pressure part, the hysteresis ring in the desorption curve indicated not only the presence of intermediate pores, but also the presence of capillary condensation and multilayer adsorption on mesoporous and macro-porous surfaces. The hysteresis ring belongs to the H_3_ type, indicating that the material has long and narrow slit holes. This explains that the three-dimensional network structure of nanocellulose aerogel is formed by the self-aggregation of nanocellulose through hydrogen bonding. Figure 7b shows the pore size distribution of the CNF/nano-lignin aerogel obtained by the BJH method, which was consistent with the morphological characteristics.

Table 1 displays that the density of the CNF/nano-lignin aerogel gradually increased with the increasing amount of lignin nanoparticle. The mechanical compression strength of the composite aerogel reached the maximum when the addition amount was 13.5 wt%, while the specific surface area of the aerogel first increased and then decreased with the increase in the lignin nanoparticles content, reaching a maximum of 18.73 m^2^/g.

## 4. Conclusions

Nanocellulose aerogel is a kind of green adsorption material with a wide application potential. However, due to the characteristics of random crosslinking of precursor scaffolds and natural hydrophilic surface hydroxyl group, this kind of aerogel has a poor stability. An inorganic filler or organic polymer were used to improve the stability of the material, but the original natural green properties were lost and the biocompatibility of the material was reduced in the reported research. In this study, the synthesis of lignin quantitative doping CNF aerogels was experimentally investigated. The impact of various parameters (lignin content, temperature, and matrix concentration) on the property of the as-prepared materials was systematically explored, revealing the optimum conditions (13.5%, 160 °C, 1.5 wt%). The lignin content relevant to the mechanical stability and hydrophobic property of the aerogels were systematically explored and the CNF/nano-lignin hybrid aerogels were successfully prepared via a liquid nitrogen freeze-drying combined with vacuum oven drying. They exhibited a mesoporous network structure and the compressive strength of the 160–13.5% C/L-aerogel under 85% strain was 0.913 MPa. This work not only contributes to the knowledge of lignin-containing cellulose nanofibers, providing a new perspective for the utilization of lignin components, but it also does not violate the natural green properties of cellulose aerogels as adsorption materials.

## Figures and Tables

**Table 1:** Lignin content, density, mechanical properties, and specific surface area of CNF/nano-lignin aerogels and CNF aerogel.

Materials	Lignin Content (wt%)	Density (g/cm^3^)	Compressive Modulus (MPa)	S_BET_ (m^2^/g)
CNF-Aerogel	0	0.01312	0.20717	12.59
160-C/L-Aerogel-1	4.5	0.01448	0.55628	16.03
160-C/L-Aerogel-2	9.0	0.01504	0.74157	18.73
160-C/L-Aerogel-3	13.5	0.01665	0.91319	16.04
160-C/L-Aerogel-4	18.0	0.01776	0.75849	14.31

## Data Availability

No new data were created or analyzed in this study. Data sharing is not applicable to this article.
